# Environmental justice index and prevalence of asthma and COPD in US neighborhoods- a population-based study

**DOI:** 10.1016/j.lana.2025.101195

**Published:** 2025-08-05

**Authors:** Sumanth Khadke, Vidhatri Khadke, Ashish Kumar, Bhargav Makwana, Sourbha S. Dani, Sadeer Al-Kindi, Sanjay Rajagopalan, Yixin Kong, Khurram Nasir, Gary Adamkiewicz, Anju Nohria, Timothy N. Liesching, Sarju Ganatra, Victor Pinto-Plata

**Affiliations:** a Division of Cardiovascular Medicine, Department of Internal Medicine, Lahey Hospital & Medical Center, 41 Mall Road, Burlington, MA, USA; b Center for Bioinformatics and Functional Genomics, Department of Biomedical Sciences, Cedars-Sinai Medical Center, Los Angeles, CA, 90048, USA; c Department of Cardiology, Mayo Clinic, Rochester, USA; d Center for Health and Nature, and the Division of Cardiovascular Prevention and Wellness, Houston Methodist, Houston, TX, USA; e Harrington Heart and Vascular Institute, University Hospitals and Case Western Reserve School of Medicine, Cleveland, OH, USA; f Department of Environmental Health, Harvard T.H. Chan, School of Public Health, Boston, MA, USA; g Cardiovascular Division, Brigham and Women's Hospital, Boston, MA, USA; h Division of Pulmonary Medicine, Department of Medicine, Lahey Hospital & Medical Center, Burlington, MA, USA

**Keywords:** Environmental justice index (EJI), Social vulnerability index, Environmental burden index, Social determinants of health (SDOH), Asthma and COPD

## Abstract

**Background:**

The independent effects of social and environmental factors on asthma and chronic obstructive pulmonary disease (COPD) are well-documented, but less is known about their combined impact across US neighborhoods. This study aimed to determine the combined and individual associations of neighborhood-level social vulnerability and environmental burden with the prevalence of asthma and COPD.

**Methods:**

This cross-sectional study analyzed 71,677 US census tracts, linking the 2022 CDC Environmental Justice Index (EJI) rankings and its subcomponents (environmental burden module [EBM] and social vulnerability module [SVM]) to the 2023 CDC PLACES dataset. Multivariable quasi-Poisson regression with an offset function was used to compare covariate-adjusted risk ratios of health indicators across quartiles of neighborhood socio-environmental burden.

**Findings:**

Among the 71,677 neighborhoods studied, the median proportion of females was 50.90%. The median proportions of individuals aged 18 to 44, 45 to 64, and ≥65 were 30.6%, 26.7%, and 15.3%, respectively, with 22.6% of the Hispanic population. Asthma and COPD prevalence rates increased with increasing EJI and EBM quartiles. Neighborhoods with the highest socio-environmental burden (Q4 EJI) had significantly higher rates of asthma (RR:1.102, 95% CI: 1.087–1.117, p < 0.001) and COPD (RR:1.156, 95% CI:1.141–1.172, p < 0.001) compared to neighborhoods with the lowest burden (Q1 EJI), after adjusting for covariates. Similarly, neighborhoods with the highest environmental burden (Q4 EBM) had higher rates of asthma (RR: 1.091, 95% CI: 1.064–1.118, p < 0.001) and COPD (RR:1.099, 95% CI: 1.070–1.129, p < 0.001) compared with Q1 EBM, after adjusting for SVM and other covariates.

**Interpretation:**

A higher prevalence of obstructive lung disease is associated with neighborhoods experiencing high cumulative socio-environmental burden. Environmental burden showed an independent association with asthma and COPD prevalence, even after adjusting for social vulnerability and other factors.

**Funding:**

None.

**Research in context**

**Evidence before this study**

We conducted a comprehensive literature search using PubMed, Embase, Web of Science, and Google Scholar databases from January 2010 to December 2023, without language restrictions. Search terms included combinations of “environmental justice,” “social vulnerability,” “environmental burden,” “air pollution,” “asthma,” “COPD,” “respiratory health,” “neighborhood,” and “census tract.” We included observational studies examining associations between environmental exposures and/or social factors with respiratory outcomes at the community level. Studies were excluded if they focused solely on individual-level exposures, used only county-level or broader geographic units, or lacked adequate adjustment for confounding variables. The evidence consistently demonstrated associations between single environmental exposures (particularly PM2.5) and respiratory outcomes, with meta-analyses showing pooled relative risks of 1.07–1.21 for asthma and 1.15–1.35 for COPD per interquartile range increase in air pollution exposure. Studies of social vulnerability showed stronger associations, with pooled estimates indicating 1.3–2.0-fold higher respiratory disease rates in the most vs. least socially vulnerable areas. However, few studies examined comprehensive environmental burden beyond air pollution, and none assessed the combined and interactive effects of multiple environmental exposures with social vulnerability at the neighborhood level across the entire United States.

**Added value of this study**

This study provides the first nationwide analysis of cumulative environmental burden's independent contribution to respiratory health disparities at the neighborhood level. We demonstrate that environmental factors maintain significant associations with asthma and COPD prevalence (8–11% increased risk) even after adjusting for social vulnerability, and that environmental and social factors interact synergistically rather than additively. By analyzing 17 environmental indicators across 71,677 census tracts, we quantify how comprehensive environmental burden—not just air pollution—drives respiratory health inequities and show that communities experiencing both high environmental burden and social vulnerability face multiplicative health risks.

**Implications of all the available evidence**

Respiratory health disparities cannot be effectively addressed through single-factor interventions. It is essential to understand the synergistic relationship between environmental and social factors necessitating the integrated approaches that simultaneously address environmental hazards and socioeconomic vulnerabilities of communities.

## Introduction

Asthma and chronic obstructive pulmonary disease (COPD) exhibit complex patterns of racial and ethnic disparities driven by the interplay between neighborhood-level environmental exposures and social vulnerability.^1^^,^^2^ These disparities largely stem from the historic clustering of adverse social and environmental factors in marginalized neighborhoods, a consequence of systemic racism and discriminatory policies like redlining affecting respiratory health.^3^^,^^4^ Studies have revealed that environmental vulnerability explained over 40% of the variance in pediatric asthma emergency department visits across major metropolitan areas and 40% of observed disparities in COPD outcomes.^3^ These neighborhood-level influences are further exemplified by other studies, such as the CARDIA lung study, which demonstrated that residence in socioeconomically deprived neighborhoods during young adulthood (ages 28–40) was associated with accelerated decline in lung function and higher odds of emphysema over 20 years of follow-up, independent of individual socioeconomic status and smoking behaviors.^1^

1ptThe evidence from previous studies collectively demonstrates strong associations between neighborhood-level factors, environmental exposures, and respiratory health outcomes, particularly asthma. Tyris et al. found that census tracts with higher Child Opportunity Index scores, especially in health/environmental and social/economic domains, were associated with lower emergency department visit rates for pediatric asthma.^5^ Similarly, Zanobetti et al. showed that early-life exposure to PM2.5 and NO2 air pollution was associated with increased asthma incidence, with higher risk among minoritized families living in urban communities with fewer opportunities and resources.^6^ This aligns with findings from Aris et al. (2023) that neighborhood characteristics at birth, including higher population density and poverty, were associated with elevated asthma risk, with Black and Hispanic children showing consistently higher risk across all neighborhood types.^7^

While previous analyses have relied on broader county-level data or individual healthcare outcomes, studying nationwide environmental and social factors at the census tract level provides crucial, granular resolution for understanding the burden of asthma and COPD.^8^^,^^9^ Environmental exposures, notably air pollution, can trigger immediate respiratory effects and induce lasting epigenetic modifications affecting gene expression patterns across generations.^10^ These biological changes may be amplified in socially vulnerable communities where residential segregation has historically concentrated environmental hazards and social stressors.^10^

Although the individual associations of social factors and environmental exposures with asthma and COPD are well established, the impact of their combined effects, and crucially, the contribution of environmental factors beyond social determinants, remains poorly understood. This knowledge gap is critical because the relationship between social and environmental disadvantages and their relative contributions to respiratory health inequities is complex and nuanced. For instance, if environmental exposures primarily mediate the relationship between social disadvantage and respiratory health outcomes (or vice versa), interventions targeting either factor could improve health disparities. However, without understanding these interactions, opportunities for effective intervention may be missed.

This study investigates the relationship between the Environmental Justice Index (EJI), which incorporates 17 different environmental indicators alongside social vulnerability measures, and the prevalence of asthma and COPD across US census tracts. This granular geographic analysis can help identify communities where targeted interventions may be most effective in addressing respiratory health disparities by analyzing both the combined and independent effects of social and environmental factors. Additionally, we explore the incremental impact of environmental factors, beyond social determinants, on asthma and COPD prevalence to better inform comprehensive policy approaches to reducing respiratory health inequities.

## Methods

This cross-sectional study analyzed data from 71,677 US census tracts using the Centers for Disease Control and Prevention (CDC) Environmental Justice Index.^11^ The study adhered to the Strengthening the Reporting of Observational Studies in Epidemiology (STROBE) guidelines for reporting cross-sectional research. Institutional review board approval was not required, as the study utilized publicly available, de-identified data.

### Data sources and measures

Asthma and COPD prevalence among adults aged 18 years and above was extracted from the 2023 CDC PLACES dataset, based on the 2020–2021 Behavioral Risk Factor Surveillance System (BRFSS), using a validated multilevel regression and poststratification approach for small-area estimation.^12^^,^^13^ Computation of the prevalence of asthma and COPD at the census tract level has been described in the Supplemental Methods section.

### Environmental justice index

The Environmental Justice Index (EJI) assesses the cumulative environmental burden on health equity at the census tract level, advancing upon previous screening and mapping tools, such as CalEnviroScreen and the US Environmental Protection Agency's (EPA's) EJSCREEN. It utilizes data from multiple federal agencies to evaluate environmental injustice across more than 71,000 US census tracts.

**Fig. 1:**
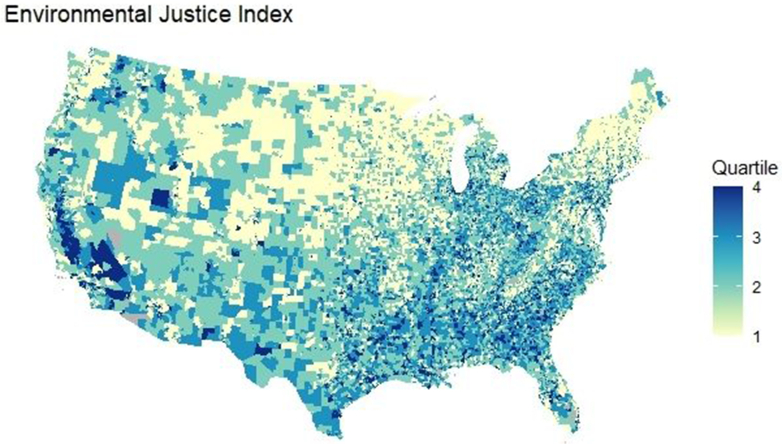
Choropleth maps of the Environmental Justice Index across census tracts. **Abbreviation:** EJI: Environmental Justice Index; COPD: Chronic obstructive pulmonary disease. EJI categories were defined based on ranked percentile scores: low (<25th percentile), medium (51st–75th percentile), and high (76th–100th percentile). EJI combines both environmental and social vulnerability measures.

**Fig. 2:**
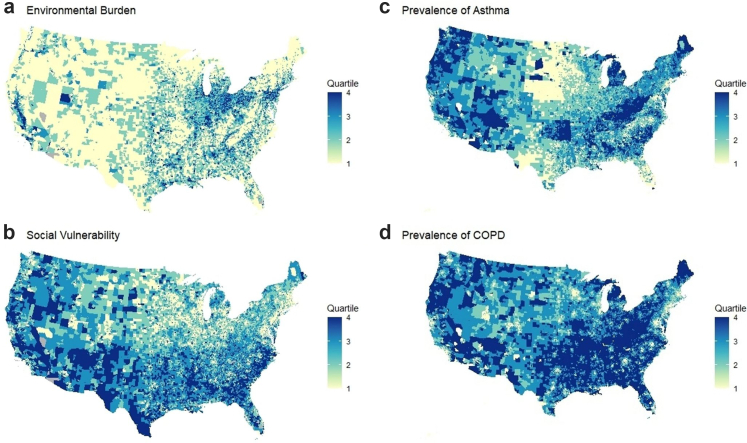
Choropleth maps of environmental burden, social vulnerability, and pulmonary outcomes across the US Census tracts. a) the US choropleth map of environmental burden at the census tract level. b) the US choropleth map of social vulnerability at the census tract level. c) the US choropleth map of the prevalence rate of asthma at the census tract level. d) the US choropleth map of COPD prevalence at the census tract level. Created using R programming. Source: CDC PLACES database. **Abbreviation:** EBM: Environmental burden module; SVM: Social vulnerability module; COPD: Chronic obstructive pulmonary disease. Environmental burden categories were defined based on ranked percentile scores of the Environmental Burden Module (EBM): low medium (25th–50th percentile), medium (51st–75th percentile), and high (76th–100th percentile). EBM includes measures of air quality, built environment, and other environmental hazards. Social vulnerability categories were defined based on ranked percentile scores of the Social Vulnerability Module (SVM): low medium (25th–50th percentile), medium (51st–75th percentile), and high (76th–100th percentile). SVM includes measures of socioeconomic status, minority status, housing, and transportation. Table represents two distinct analyses: (1) Association of environmental burden module (EBM) quartiles with asthma and COPD prevalence, adjusted for social vulnerability module (SVM) in three scenarios: unadjusted, adjusted for age categories only, and fully adjusted for multiple covariates; (2) Association of social vulnerability module (SVM) quartiles with asthma and COPD prevalence, adjusted for environmental burden module (EBM) in the same three scenarios. EBM-SVM interaction was tested in all models. Fully adjusted models include age category, healthcare visits, gender percentage, smoking status, and rurality as covariates. RR = relative risk; CI = confidence interval.

The EJI model ranks each census tract based on 36 environmental, social, and health factors, categorized into three overarching modules and ten distinct domains. The overall EJI score combines the Environmental Burden Module, Social Vulnerability Module, and Health Vulnerability Module. This study focuses on the socio-environmental burden (excluding the Health Vulnerability component), as shown in Fig. 1. In this manuscript, we refer to the socio-environmental burden when referring to the EJI as shown in Fig. 2a.

### Environmental burden module

The Environmental Burden Module (EBM), released in 2022, comprehensively measures environmental factors that affect health. It utilizes data from 2014 to 2021 from various federal agencies, encompassing 17 indicators across five domains: air pollution, hazardous sites, built environment, transportation, and water pollution. Each indicator is a percentile rank, with domain scores calculated by summing indicators and converting sums to percentile scores. The overall EBM aggregates all indicators into a score ranging from 0 to 1, with higher scores indicating a greater environmental burden. The Supplemental Table S1 details the EBM indicators.

### Social vulnerability module

Neighborhood-level social vulnerability module (SVM) data were obtained from the CDC. The SVM module measures the demographic and socioeconomic factors influencing community resilience to health stresses. The social vulnerability index (SVM) encompasses 14 indicators across four domains using 2015–2019 data: (1) racial/ethnic minority status, (2) socioeconomic status (e.g., poverty, unemployment), (3) household characteristics (e.g., disability, limited English fluency), and (4) housing type (e.g., group quarters). Each indicator consists of a percentile rank. Analogous to the EBM, the overall SVM sums component indicators and then converts the total to a percentile score at the neighborhood level, ranging from 0 to 1. Higher scores denote higher social vulnerability, as shown in Fig. 2b, Supplemental Table S1. Lists the details of the SVM indicators.

Additional covariates included age and sex composition, smoking prevalence, health checkup rates, and rural-urban classification.

### Outcomes

We assessed the prevalence of asthma and COPD, both defined by self-reported clinical diagnoses.

### Statistical analysis

We divided neighborhoods into quartiles based on their EJI scores, with quartile 4 (Q4) representing the highest socio-environmental burden and quartile 1 (Q1) representing the lowest burden. A similar quartile-based division was repeated with EBM and SVM scores. Neighborhood characteristics and covariates were summarized descriptively and compared across EJI quartiles using χ2 and Kruskal–Wallis tests.

We conducted analyses to examine two key questions: 1) the relationship between EJI quartiles and respiratory disease prevalence, and 2) the independent association of environmental burden (EBM) with respiratory outcomes, after adjusting for social vulnerability (SVM) and other covariates. The principal analysis was conducted by fitting multivariable quasi-Poisson regression models with an offset function to the neighborhood's total population log to evaluate relative associations across the quartiles of socio-environmental burden at the neighborhood level. Models were adjusted for neighborhood age and sex composition, SVM quartile, smoking status, rurality, and the percentage of adults who received routine checkups the previous year. SVM adjustment was omitted when EJI was used as an exposure. We also repeated this principal analysis after including the continuous SVM, apart from the SVM quartiles, as a covariate in our models.

EBM × SVM interaction terms were included in our models to assess whether associations between environmental burden and pulmonary outcomes varied across different levels of neighborhood social vulnerability. The environmental burden was treated as a continuous exposure in these models to improve visualization of potential effect modification across different levels of neighborhood social vulnerability. To explore the potential association of distinct environmental burden domains at different levels of neighborhood social vulnerability, we conducted multiple analyses: 1) unadjusted models, 2) models adjusted for age categories and SVM only, and 3) fully adjusted models incorporating age and sex composition, healthcare visits, smoking status, and rurality as covariates. This stepwise approach allowed us to examine how the associations between environmental burden domains and respiratory outcomes changed with progressive adjustment for potential confounders. All statistical tests were 2-sided, and p < 0.05 was considered statistically significant. Analyses were conducted between October 2023 and June 2024. We used the R Studio 2023.09.01 version for statistical analysis.

### Role of funding source

This study has not received any external funding.

## Results

### Demographics

Our study sample included 71,677 census tracts encompassing a total population of approximately 320 million individuals across the US. The population was evenly distributed across both the Environmental Justice Index (EJI) quartiles and the Environmental Burden Module (EBM) quartiles. EJI Q1 (lowest burden) contained 17,920 tracts, and Q4 (highest burden) contained 17,924 tracts. Similarly, EBM Q1 and Q4 included 17,928 and 17,901 tracts, respectively. The geographic distribution of neighborhood socio-environmental burden is illustrated in Fig. 1.

### Age and sex

EJI Quartiles: In Q4-EJI neighborhoods (with the highest socio-environmental burden), the population had a higher proportion of younger adults aged 18–44 [n = 23,664,189 (33.26%)] compared to Q1-EJI [n = 23,175,354 (29.6%)]. Older adults were more concentrated in Q1-EJI: ages 45–64 totaled 22,057,867 (29.6%) and ≥65 totaled 12,504,493 (16.7%), vs. 16,473,094 (23.9%) and 8,830,017 (12.2%), respectively, in Q4-EJI. The median female percentage increased across quartiles, from 50.61% in Q1 to 51.25% in Q4.

EBM Quartiles: A similar trend was seen across environmental burden levels. Q4-EBM neighborhoods (with the highest environmental burden) had 24,738,364 (32.8%) individuals aged 18–44, compared to 24,815,364 (30.6%) in Q1-EBM. However, Q1-EBM neighborhoods had a higher proportion of older adults: 22,047,858 (28.1%) aged 45–64 and 13,936,869 (17.2%) aged ≥65, compared to 18,437,598 (25.2%) and 10,067,702 (13.2%) in Q4-EBM. Median female percentage was 50.61% in Q1-EBM and 51.10% in Q4-EBM.

### Race and ethnicity

In the EJI Quartiles, racial and ethnic disparities were marked. Q4-EJI neighborhoods had 17,533,628 (29.31%) Black residents and 22,725,621 (48.04%) Hispanic residents, compared to only 3,672,701 (4.97%) and 6,448,973 (9.65%) in Q1-EJI, respectively. White individuals comprised 37,911,097 (63.38%) of the Q4-EJI population vs. 64,926,234 (87.98%) in Q1-EJI [Table 1].

**Table 1:** Demographic distribution of the U.S. population stratified across the Environmental justice index quartiles.

Socio-environmental burden-EJI quartiles						
Demographics	All neighborhoods (N = 71,677)	First (lowest) (N = 17,920)	Second (N = 17,913)	Third (N = 17,920)	Fourth (highest) (N = 17,924)	p value
Total population	320,286,908	83,841,822	81,889,031	80,722,330	73,833,725	
Percent female median (IQR)	50.90 (50.87–50.93)	50.61 (48.82–52.42)	50.76 (48.84–52.74)	51.11 (48.92–53.32)	51.25 (48.75–53.89)	<0.001
Age						
Age 18–44	99,099,193	23,175,354	22,989,249	23,263,209	23,664,189	<0.001
Median Percent 18–44 (Median IQR)	30.64 (18.70–48.90)	29.60 (17.84–48.10)	30.02 (18.21–47.30)	29.94 (18.60–47.45)	33.26 (20.25–52.45)	<0.001
Age 45–64	83,324,737	22,057,867	20,294,329	18,963,552	16,473,094	<0.001
Median percent 45–64 (Median IQR)	26.7 (22.9–30.1)	29.6 (26.1–32.6)	27.6 (24.1–30.6)	25.9 (22.4–28.9)	23.9 (20.9–27.0)	<0.001
Age ≥ 65	48,912,343	12,504,493	12,169,159	11,334,840	8,830,017	<0.001
Median percent ≥65 (Median IQR)	15.3 (11–19.6)	16.7 (12.50–21.22)	16.5 (12.5–20.6)	15.3 (11.2–19.4)	12.2 (9.0–16.4)	<0.001
Race No. (%)						
Whites	234,060,614 (79.36%)	64,926,234 (87.98%)	60,937,877 (84.93%)	54,536,649 (78.22%)	37,911,097 (63.38%)	<0.001
Blacks	40,860,717 (13.85%)	3,672,701 (4.97%)	5,917,841 (8.24%)	10,294,539 (14.76%)	17,533,628 (29.31%)	<0.001
American Indian or Alaskan Native	2,589,239 (0.87%)	373,459 (0.50%)	842,608 (1.17%)	747,137 (1.07%)	548,735 (0.91%)	<0.001
Asian	16,990,644 (5.76%)	4,731,615 (6.41%)	3,946,621 (5.50%)	4,031,158 (5.78%)	3,702,080 (6.19%)	<0.001
Native Hawaiian or Other Pacific Islander	430,518 (0.14%)	90,863 (0.12%)	97,619 (0.13%)	110,810 (0.15%)	117,201 (0.19%)	<0.001
Ethnicity No. (%)						
Hispanic	57,316,166 (22.60%)	6,448,973 (9.65%)	9,033,620 (14.17%)	13,834,849 (23.36%)	22,725,621 (48.04%)	<0.001
Non-hispanic	196,407,478 (77.40%)	60,370,942 (90.34%)	54,720,371 (85.83%)	45,371,239 (76.63%)	24,580,198 (51.96%)	<0.001
Lack of insurance						
Median (IQR)	9200 (6400–14000)	6200 (4700–7900)	7900 (6000–11100)	9900 (7400–14200)	15,500 (11,500–21400)	<0.001
Rurality No. (%)						
Urban neighborhoods	59,804 (83.4%)	15,234 (85%)	13,908 (77.64%)	14,416 (80%)	16,246 (90.6%)	<0.001
Rural neighborhoods	11,873 (16.6%)	2686 (15%)	4005 (22.36%)	3504 (20%)	1678 (9.4%)	<0.001

In Q4-EBM neighborhoods, the number of Black residents was 14,017,833 (21.32%) and Hispanic residents totaled 18,271,895 (33.6%), compared to 5,661,363 (7.41%) and 12,796,993 (18.52%) in Q1-EBM. White populations declined from 65,776,628 (86.18%) in Q1-EBM to 46,728,392 (71.10%) in Q4-EBM [Table 2].

**Table 2:** Demographic distribution of the U.S. population stratified across the Environmental burden index quartiles.

Environmental burden module quartiles						
Demographics	All neighborhoods (N = 71,677)	First (lowest) (N = 17.928)	Second (N = 17,926)	Third (N = 17,922)	Fourth (highest) (N = 17,901)	p value
Total population	320,286,908	81,322,605	83,492,777	81,067,500	74,404,026	
Percent female Median (IQR)	50.90 (50.87–50.93)	50.61 (48.68–52.57)	50.82 (48.83–52.87)	51.13 (48.99–53.33)	51.10 (48.84–53.47)	<0.001
Age						
Age 18–44	99,099,193	24,815,364	24,714,445	24,616,239	24,738,364	<0.001
Median Percent 18–44 (Median IQR)	30.64 (18.70–48.90)	30.60 (18.32–49.19)	29.11 (17.90–46.48)	30.04 (18.44–47.71)	32.87 (20.20–52.18)	<0.001
Age 45–64	83,324,737	22,047,858	21,989,968	20,778,533	18,437,598	<0.001
Median Percent 45–64 (Median IQR)	26.7 (22.9–30.1)	28.1 (24.4–31.5)	27.1 (23.4–30.4)	26.3 (22.6–29.6)	25.2 (21.6–28.7)	<0.001
Age ≥65	48,912,343	13,936,869	13,000,780	11,898,096	10,067,702	<0.001
Median Percent ≥65 (Median IQR)	15.3 (11–19.6)	17.2 (12.6–22.2)	16.0 (11.8–20.0)	14.8 (10.8–18.7)	13.2 (9.5–17.4)	<0.001
Race -No. (%)						
Whites	234,060,614 (79.36%)	65,776,628 (86.18%)	64,076,280 (82.19%)	57,261,788 (76.81%)	46,728,392 (71.10%)	<0.001
Blacks	40,860,717 (13.85%)	5,661,363 (7.41%)	9,005,144 (11.55%)	12,051,195 (16.16%)	14,017,833 (21.32%)	<0.001
American Indian or Alaskan Native	2,589,239 (0.87%)	1,116,294 (1.46%)	619,799 (0.79%)	460,745 (0.61%)	386,490 (0.58%)	<0.001
Asian	16,990,644 (5.76%)	3,646,036 (4.77%)	4,140,019 (5.31%)	4,670,156 (6.26%)	4,515,314 (6.86%)	<0.001
Native Hawaiian or Other Pacific Islander	430,518 (0.14%)	119,420 (0.15%)	113,407 (0.14%)	102,803 (0.14%)	93,750 (0.14%)	<0.001
Ethnicity-No. (%)						
Hispanic No. (%)	57,316,166 (22.60%)	12,796,993 (18.52)	12,472,705 (18.36%)	13,683,251 (22.00%)	18,271,895 (33.6%)	<0.001
Non-hispanic No. (%)	196,407,478 (77.40%)	56,296,564 (81.48%)	55,450,401 (81.63%)	48,514,042 (78.00%)	35,983,554 (66.3%)	<0.001
Lack of insurance						
Median (IQR)	9200 (6400–14000)	8100 (6000–12000)	8600 (6100–12800)	9100 (6400–13900)	11,400 (7500–17400)	<0.001
Rurality No. (%)						
Urban neighborhoods	59,804 (83.4%)	13,010 (72.6%)	14,235 (79.4%)	15,702 (87.6%)	16,858 (94.2%)	<0.001
Rural neighborhoods	11,873 (16.6%)	4919 (27.4%)	3691 (20.6%)	2220 (12.4%)	1043 (5.8%)	<0.001

### Insurance and urbanicity

In EJI quartiles, the median number of uninsured individuals per 100,000 was 6200 in Q1-EJI and rose to 15,500 in Q4-EJI. Urban tracts increased from 15,234 (85%) in Q1-EJI to 16,246 urban tracts (90.6%) in Q4-EJI [Table 1].

In EBM quartiles, the insurance disparities also persisted across EBM quartiles. Q1-EBM had a median uninsured rate of 8100 per 100,000, whereas Q4-EBM had a rate of 11,400 per 100,000. Urban tracts accounted for 72.6% (n = 13,010) in Q1-EBM and rose sharply to 94.2% (n = 16,858) in Q4-EBM [Table 2].

### Neighborhood socio-environmental burden and prevalence of asthma and COPD

In neighborhoods within the highest quartile of socio-environmental burden (Q4-EJI), the mean prevalence of asthma was 11,488 cases per 100,000 population (SD: 1651.79), compared to 9684 per 100,000 (SD: 984.9) in the lowest quartile (Q1-EJI). This corresponds to an adjusted relative risk (RR) of 1.102 (95% CI: 1.087–1.117; p < 0.001), indicating a 10.2% higher asthma prevalence in the most burdened communities after adjusting for covariates, including age, sex, smoking, healthcare access, rurality, and social vulnerability. The unadjusted RR was 1.350 (95%CI: 1.333–1.368; p < 0.001) [Fig. 2c, Supplemental Table S1 and Table 3].

**Table 3:** Unadjusted and adjusted regression model demonstrating the association of Socio-Environmental burden and pulmonary diseases.

Socio-environmental Burden	Unadjusted models			Age category adjusted models			Age category, health care visits, gender percentage, Smoking and rurality- adjusted model		
	Low medium EJI	Medium EJI	High EJI	Low medium EJI	Medium EJI	High EJI	Low medium EJI	Medium EJI	High EJI
Asthma	1.073 (1.059–1.088), p < 0.001	1.132 (1.117–1.148), p < 0.001	1.350 (1.333–1.368), p < 0.001	1.089 (1.075–1.103), p < 0.001	1.174 (1.159–1.189), p < 0.001	1.451 (1.433–1.470), p < 0.001	0.985 (0.973–0.996), p = 0.013	0.996 (0.984–1.009), p = 0.610	1.102 (1.087–1.117), p < 0.001
COPD	1.223 (1.201–1.245), p < 0.001	1.333 (1.310–1.357), p < 0.001	1.669 (1.640–1.698), p < 0.001	1.283 (1.263–1.304), p < 0.001	1.491 (1.468–1.516), p < 0.001	2.068 (2.035–2.102), p < 0.001	1.021 (1.009–1.034), p < 0.001	1.046 (1.034–1.059), p < 0.001	1.156 (1.141–1.172), p < 0.001

EJI: Environmental Justice Index; COPD: Chronic obstructive pulmonary disease. EJI categories were defined based on ranked percentile scores: low medium (25th–50th percentile), medium (51st–75th percentile), and high (76th–100th percentile). EJI combines both environmental and social vulnerability measures.

### Neighborhood environmental burden and prevalence of asthma and COPD

Neighborhoods with the highest environmental burden alone (EBM) had a mean asthma prevalence of 10,845.5 per 100,000 (SD 1688.1), while Q1-EBM neighborhoods had 10,180.1 per 100,000 (SD 1195.04). After full covariate adjustment, asthma prevalence remained significantly elevated in Q4-EBM neighborhoods with an adjusted RR of 1.091 (95% CI: 1.064–1.118; p < 0.001), confirming an independent effect of environmental burden on asthma rates. The unadjusted RR was 1.151 (1.135–1.166; p < 0.001) [Supplemental Table S2 and Table 4].

**Table 4:** Unadjusted and adjusted regression model demonstrating the association of Environmental burden and pulmonary diseases.

	Unadjusted models			SVM and age category adjusted model			SVM, age category, health care visits, gender percentage, smoking, and rurality-adjusted model		
	Low medium EBM	Medium EBM	High EBM	Low Medium EBM	Medium EBM	High EBM	Low Medium EBM	Medium EBM	High EBM
Asthma	0.978 (0.964–0.991), p = 0.001	1.027 (1.013–1.041), p < 0.001	1.151 (1.135–1.166), p < 0.001	0.987 (0.975–1.000), p = 0.053	1.030 (1.018–1.043), p < 0.001	1.126 (1.112–1.140), p < 0.001	0.991 (0.970–1.013), p = 0.459	1.000 (0.978–1.023), p = 0.963	1.091 (1.064–1.118), p < 0.001
COPD	0.958 (0.941–0.97), p < 0.001	0.989 (0.971–1.007), p = 0.25	1.118 (1.099–1.113), p < 0.001	1.041 (1.010–1.074), p = 0.0079	1.073 (1.039–1.107), p < 0.001	1.154 (1.114–1.196), p < 0.001	1.020 (0.997–1.044), p = 0.086	1.032 (1.007–1.057), p = 0.010	1.099 (1.070–1.129), p < 0.001

SVM categories	Unadjusted models			EBM and age category adjusted model			EBM, age category, health care visits, gender percentage, smoking, and rurality-adjusted model		
	Low medium SVM	Medium SVM	High SVM	Low medium SVM	Medium SVM	High SVM	Low medium SVM	Medium SVM	High SVM
Asthma	1.118 (1.103–1.134), p < 0.001	1.224 (1.207–1.240), p < 0.001	1.402 (1.384–1.421), p < 0.001	1.123 (1.109–1.137), p < 0.001	1.251 (1.235–1.267), p < 0.001	1.470 (1.451–1.490), p < 0.001	1.013 (0.991–1.036), p = 0.222	0.985 (0.962–1.008), p = 0.212	0.951 (0.925–0.978), p < 0.001
COPD	1.349 (1.325–1.374), p < 0.001	1.656 (1.627–1.685), p < 0.001	2.013 (1.978–2.048), p < 0.001	1.465 (1.423–1.508), p < 0.001	1.945 (1.890–2.001), p < 0.001	2.383 (2.308–2.460), p < 0.001	1.095 (1.071–1.120), p < 0.001	1.121 (1.095–1.147), p < 0.001	1.098 (1.070–1.127), p < 0.001

EBM: Environmental burden module; SVM: Social vulnerability module; COPD: Chronic obstructive pulmonary disease. Environmental burden categories were defined based on ranked percentile scores of the Environmental Burden Module (EBM): low medium (25th–50th percentile), medium (51st–75th percentile), and high (76th–100th percentile). EBM includes measures of air quality, built environment, and other environmental hazards. Social vulnerability categories were defined based on ranked percentile scores of the Social Vulnerability Module (SVM): low medium (25th–50th percentile), medium (51st–75th percentile), and high (76th–100th percentile). SVM includes measures of socioeconomic status, minority status, housing, and transportation. Table represents two distinct analyses: (1) Association of environmental burden module (EBM) quartiles with asthma and COPD prevalence, adjusted for social vulnerability module (SVM) in three scenarios: unadjusted, adjusted for age categories only, and fully adjusted for multiple covariates; (2) Association of social vulnerability module (SVM) quartiles with asthma and COPD prevalence, adjusted for environmental burden module (EBM) in the same three scenarios. EBM-SVM interaction was tested in all models. Fully adjusted models include age category, healthcare visits, gender percentage, smoking status, and rurality as covariates. RR = relative risk; CI = confidence interval.

For COPD, Q4-EJI neighborhoods exhibited a mean prevalence of 8130 cases per 100,000 (SD: 2733.72), compared to 5546 per 100,000 (SD: 1818.06) in Q1-EJI neighborhoods. This reflects an adjusted RR of 1.156 (95% CI: 1.141–1.172; p < 0.001), indicating a 15.6% higher prevalence in the most burdened areas. The unadjusted RR was 1.669 (95% CI:1.640–1.698; p < 0.001) [Supplemental Table S2 and Table 3].

Under environmental burden stratification, Q4-EBM neighborhoods had a mean COPD prevalence of 7060.3 per 100,000 (SD: 2793.6), vs. 6292 per 100,000 (SD: 2469.8) in Q1-EBM neighborhoods. The adjusted RR for COPD in Q4-EBM was 1.099 (95% CI: 1.070–1.129; p < 0.001), and unadjusted RR was 1.118 (95%CI: 1.099–1.113; p < 0.001), supporting an independent association between environmental burden and COPD prevalence even after accounting for social vulnerability and other covariates [Supplemental Table S2 and Table 4].

The severity-dependent pattern of prevalence rate ratios for asthma and COPD was consistent across all models, including those with only SVM and age category adjustment, as shown in Table 3, Table 4.

### Social vulnerability burden and prevalence of asthma and COPD

In census tracts within the lowest quartile of social vulnerability (Q1-SVM), the mean asthma prevalence was 9455 cases per 100,000 population (SD: 885.2), compared to the highest quartile having 11,676 per 100,000 (SD: 1579.5), representing an absolute increase of over 2200 cases per 100,000 population.

After adjusting for age, sex, smoking prevalence, healthcare access, rurality, and environmental burden, the adjusted relative risk (RR) for asthma in Q4-SVM vs. Q1-SVM was 0.951 (95% CI: 0.925–0.978; p < 0.001). The unadjusted RR was 1.235 (95% CI: 1.219–1.252; p < 0.001).

The mean COPD prevalence in Q1-SVM neighborhoods was 4921 cases per 100,000 (SD: 1445.6), rising to 8722 per 100,000 (SD: 2609.4) in Q4-SVM. This marks an increase of nearly 3800 cases per 100,000 from the least to the most socially vulnerable areas. The adjusted RR for COPD in Q4-SVM vs. Q1-SVM was 1.098 (95% CI: 1.070–1.127; p < 0.001), indicating a significant and independent association after controlling for environmental and demographic factors. The unadjusted RR was at 2.013 (95% CI: 1.978–2.048; p < 0.001).

The severity-dependent pattern between the census tract-level social vulnerability index and the prevalence of asthma and COPD was consistent in unadjusted, EBM, and age category-adjusted models. However, the severity-dependent pattern is not seen when robust adjustments include healthcare visits, gender, smoking, rurality, in addition to EBM and age categories, in both COPD and Asthma [Table 4]. The interaction analysis of the regression model related to social vulnerability and environmental burden using SVM∗EBM was significant (p <) in unadjusted, age-adjusted, and multiple covariate-adjusted models, as shown in Table 4.

## Discussion

In this extensive cross-sectional study of 71,677 census tracts, we found that the neighborhoods with the highest cumulative social and environmental burden (EJI) had a higher prevalence of asthma and COPD in a severity-dependent stepwise gradient. After comprehensive adjustment for neighborhood age, sex composition, smoking, and healthcare access, the highest EJI quartile showed approximately 10% higher risk of asthma and 16% higher risk of COPD compared to the lowest quartile. Environmental burden demonstrated independent associations with respiratory outcomes even after adjusting for social vulnerability, with the highest EBM quartile showing about 8% higher asthma risk and 9% higher COPD risk compared to the lowest EBM quartile. Notably, social vulnerability showed the strongest independent association among the three indices, with a 22% higher COPD risk in areas of the highest social vulnerability, although this effect attenuated somewhat after full adjustment. This study advances beyond previous research by comprehensively examining how 17 different environmental indicators interact with social vulnerability measures at the census tract level, revealing that environmental factors disproportionately affect respiratory health in socially vulnerable communities. The significant interactions observed between environmental and social factors suggest that addressing either factor alone may be insufficient; instead, the most significant reductions in respiratory health disparities may require simultaneous attention to both environmental exposures and social vulnerabilities. We have demonstrated the chronicity of environmental exposures in Supplemental Table S3.

To our knowledge, this is the first study to analyze the impact of social and environmental factors on respiratory health, including (i) the combined impact of social and environmental factors together (ii) the impact of environmental factors beyond air pollution along with exploring interaction between social vulnerability and environmental burden, (iii) additional association of environmental factors with pulmonary health after accounting for social determinants of health.

### Social and environmental interactions

Our findings reveal significant positive interactions between high environmental burden (Q4 EBM) and high social vulnerability (Q4 SVM), suggesting these factors act synergistically rather than merely additively affecting respiratory health outcomes. From a public health perspective, this synergism indicates that communities facing both high environmental exposures and social vulnerability experience a multiplicative rather than additive burden of respiratory disease. For example, while high environmental burden alone was associated with a 9.3% increased risk of COPD and high social vulnerability alone with a 22.1% increased risk, communities experiencing both showed a substantially higher risk than would be expected from combining these independent effects. This has critical implications for population health strategies: interventions targeting either environmental exposures or social vulnerability alone may yield suboptimal results compared to comprehensive approaches that address both simultaneously. From a clinical standpoint, this interaction suggests healthcare providers should consider environmental and social contexts when assessing respiratory disease risk and managing care. Patients from communities with both a high environmental burden and social vulnerability may require more intensive preventive care, earlier screening, and more aggressive management strategies for respiratory conditions. Additionally, clinicians should consider that standard therapeutic approaches may have different efficacy in these multiply burdened populations, potentially necessitating tailored treatment strategies that account for both environmental exposures and social constraints. This synergistic relationship underscores the importance of integrating environmental and social risk assessment into clinical decision-making for respiratory health.

### Context within existing literature

Our findings build upon and extend prior studies that have primarily focused on single exposures, particularly air pollution, with most demonstrating associations between PM2.5 and respiratory outcomes. For instance, a global meta-analysis of 25 million participants revealed that every 10 μg/m3 increment in PM2.5 was associated with a 21.4% increased risk of childhood asthma and a 7.1% increased risk in adults.^14^ Studies examining COPD have shown similar patterns, with the UK Biobank study reporting a 17% increased COPD risk per interquartile range (IQR) increase in PM 2.5.^15^ Our findings align with and advance beyond recent county-level analyses of social and environmental vulnerabilities. For example, Lee et al. demonstrated that counties in the highest social vulnerability index quartile had 44% higher COPD mortality rates compared to those in the lowest quartile.^16^ While informative, their county-level analysis could not disentangle specific environmental effects. Similarly, Lotfata et al.'s work, which utilized geographically weighted random forests, identified behavioral factors, such as smoking, as primary drivers of asthma prevalence, with environmental factors playing a seemingly lesser role.^17^ In contrast, our analysis using the comprehensive EJI framework at the census tract level (geographically microscopic level compared to the county) reveals several crucial insights that these previous studies missed. First, examining single exposures or county-level data likely underestimates the actual burden of environmental health inequities. By incorporating 17 distinct environmental indicators alongside measures of social vulnerability at the census tract level, we demonstrate that the cumulative burden of multiple environmental exposures, particularly in socially vulnerable communities, has a substantially larger impact on respiratory health than previously recognized.^18^ Second, our findings reveal specific combinations of environmental exposures (like air pollution, built environment factors, and hazardous sites) that together drive respiratory disparities - insights masked in broader county-level analyses. Third, we show that environmental burden maintains significant independent associations with respiratory outcomes even after accounting for social vulnerability, suggesting that addressing environmental justice requires attention beyond social factors alone. This more comprehensive assessment helps explain why traditional single-exposure or county-level studies may have underestimated environmental contributions to respiratory health disparities.

### Mechanisms and interactions

The biological mechanisms underlying these associations likely involve multiple pathways amplified by social vulnerability. Environmental exposures, particularly air pollutants, trigger pro-inflammatory mechanisms, including bronchoconstriction, airway inflammation, and enhanced allergic responses.^19^^,^^20^ These can lead to oxidative stress and tissue injury, potentially causing lasting epigenetic modifications affecting gene expression patterns across generations.^21^^,^^22^ Our findings suggest these biological mechanisms may be amplified in socially vulnerable communities through several pathways. First, communities with high social vulnerability often face higher cumulative exposure burdens across multiple environmental hazards, leading to additive or synergistic biological effects.^23^ Second, social stressors may directly modify biological response pathways, potentially making individuals more susceptible to environmental insults through altered immune function and inflammatory responses.^24^ This is supported by our observation of significant interactions between environmental burden and social vulnerability measures, with environmental exposures showing stronger associations with respiratory outcomes in highly vulnerable communities.^22^ The strength of these interactions suggests that biological susceptibility to environmental exposures may be fundamentally modified by social context, highlighting why addressing environmental justice requires consideration of both environmental and social factors.^22^

Our findings reveal essential nuances in how environmental burden and social vulnerability interact to affect respiratory health. The observed relationship between high environmental burden and high social vulnerability suggests these factors create a compounding effect that exceeds their contributions. The strong association between environmental burden and respiratory outcomes in socially vulnerable communities suggests that identical environmental exposures may have different health impacts depending on the social context. This could be due to several factors not previously discussed: limited access to preventive care in vulnerable communities may allow environmental effects to accumulate over time; housing conditions in socially vulnerable areas may increase indoor exposure to outdoor pollutants; and economic constraints may prevent households from using air conditioning or air purifiers to mitigate environmental exposures.

In addition, the persistence of environmental associations after adjusting for social vulnerability suggests that environmental justice initiatives focused solely on social factors may be insufficient. The independent effect of environmental burden implies that direct environmental interventions—such as emissions controls, green space development, and built environment improvements—remain crucial even in communities with improving social conditions. These findings underscore the need for clinical and public health frameworks to incorporate socio-environmental metrics, such as the EJI, when assessing patient risk and allocating resources. Policymakers should prioritize investments in infrastructure, pollution mitigation, and healthcare access in the communities that bear the greatest burden.

### Clustering of neighborhood susceptibility

Our study reveals that areas with high environmental burden are often socially disadvantaged, reflecting historical racial injustice practices in the US. These practices led to the segregation of minority groups in unhealthy regions near hazardous sites, perpetuating economic inequality and systemic racism.^25^ Affected communities often lack political support and resources, facing socioeconomic challenges that impair their ability to protect their health. This includes limited access to health insurance, education, employment, and recreational spaces.^4^^,^^25^ Recent studies have shown that historical discriminatory policies, such as redlining, continue to contribute to environmental and health inequities, particularly for Black adults.^4^ These findings align with stress-related responses in vulnerable populations and highlight the need for policy changes. Additionally, long-term exposure to environmental hazards, including prenatal exposure, has a significant impact on respiratory health outcomes. In America, Latinx (particularly Puerto Rican and Dominican American) and non-Latinx Black individuals show higher asthma prevalence and severity, including higher emergency room visits, hospitalization, and mortality compared to white individuals.^21^^,^^26^ Our study found that Latinx and Black adults were more likely to be in the highest quartile of EJI.

Social determinants of health, including poverty, discrimination, and environmental hazards, have a significant impact on health outcomes. Studies show that social vulnerability factors are associated with higher rates of asthma and COPD and influence susceptibility to environmental insults.^27^ Additionally, social vulnerability strongly influences an individual's susceptibility to environmental insults via factors including poor baseline respiratory health, inadequate access to medications, suboptimal housing conditions, and limited adaptive capacity.^22^^,^^28^^,^^29^ However, the differential distribution of environmental hazards across communities contributes to and interacts with social vulnerability.^30^ Historical practices like redlining concentrated disadvantaged communities in areas with higher pollution, creating environmental injustice.^30^ While statistical models may attribute adverse respiratory effects more to social vulnerability, environmental and social factors are closely interlinked.^31^ Effective solutions must address both simultaneously, such as legislation limiting polluters near vulnerable communities and combining housing improvements with environmental remediation.^30^

### Limitations

Our study has several significant limitations. First, despite adjusting for social vulnerability, gender, and healthcare access, we were unable to account for individual-level confounders, such as occupational exposures, indoor air quality, or genetic predisposition to respiratory diseases. Second, our reliance on the CDC's multilevel regression and post-stratification analysis for neighborhood-level outcomes from BRFSS data, though validated in previous studies, may not capture rapid demographic changes in specific communities. The use of self-reported clinical diagnoses for disease prevalence introduces potential response bias and may underestimate actual disease burden, particularly in areas with limited healthcare access. Third, our environmental burden measures, while comprehensive, do not capture all potential environmental hazards. Notable exclusions include indoor air quality, seasonal variations in exposure, and emerging environmental threats. The Environmental Justice Index data represents a snapshot in time and may not reflect historical exposures that could influence current disease patterns. Fourth, while census tract-level analysis provides granular insight, it may mask within-tract variations in environmental exposure and disease burden. The ecological nature of our study means findings cannot be directly applied to individual-level risk assessment. Fifth, our study could not account for population mobility between census tracts, which may lead to exposure misclassification.

Additionally, we lack critical individual-level information, including disease duration, age of onset, allergic status, disease phenotypes, and severity classifications. The database does not contain pulmonary function measurements, inflammatory biomarkers, or detailed smoking history that would help characterize disease subtypes and severity. Additionally, the cross-sectional nature of BRFSS prevents us from understanding disease progression or determining causality. Despite these limitations, the consistency of our findings in the unadjusted and adjusted models, as well as the robust associations observed even after controlling for multiple confounders, suggests that our results reflect genuine relationships between environmental burden and respiratory disease prevalence.

### Future directions

Future research should prioritize longitudinal studies examining how neighborhood-level environmental exposures interact with social vulnerability to influence respiratory health outcomes over time. Specifically, studies should focus on: 1) tracking respiratory outcomes from early life through adulthood in communities with varying levels of environmental burden; 2) evaluating how changes in neighborhood environmental conditions affect asthma and COPD trajectories; and 3) assessing the impact of targeted environmental interventions in socially vulnerable communities.

Additionally, clinical research is needed to understand how environmental burden affects treatment response and disease management. This includes examining whether patients from high-burden neighborhoods require modified treatment approaches, developing risk-stratification tools that incorporate environmental justice indices, and evaluating the effectiveness of integrated care models that address both clinical and environmental factors. Such evidence would help inform clinical guidelines and support healthcare delivery modifications needed to reduce respiratory health disparities in environmentally overburdened communities.

### Conclusion

Neighborhoods with high socio-environmental burden demonstrated significantly higher prevalence rates of asthma and COPD compared to areas with the lowest burden. Environmental burden was found to have an independent association with respiratory disease prevalence, even after adjusting for social vulnerability. Areas with the highest environmental burden showed increased rates of asthma and COPD compared to those with the lowest burden. The severity-dependent pattern and significant interaction between environmental and social factors suggest the need for further research to understand the temporal relationship between cumulative environmental exposures and respiratory outcomes, particularly in vulnerable populations. Longitudinal studies, starting from pregnancy or birth, are needed to fully understand the effects of prolonged exposure on respiratory health outcomes. Hence, incorporating EJI and environmental burden scores into public health surveillance and clinical decision-making tools could help identify high-risk neighborhoods, tailor interventions, and inform policy reforms aimed at mitigating respiratory health disparities.

## Contributors

All authors, in adherence to the rigorous standards set by the International Committee of Medical Journal Editors (ICMJE), have meticulously participated in the research and preparation of the manuscript, ensuring the thoroughness and validity of the findings. SK, VK, VPP, and SG contributed to the study design, literature search, data collection, data analysis, and writing the original draft. VK, BM, AK, KN, RW, YK, GA, SR, TNL, and SAK supervised, validated, and reviewed the preliminary draft and assisted in editing. VPP, SG, AN, and TNL were involved in review and editing. SK and VK were involved in visualizing the data. All authors approved the final manuscript and accepted responsibility for the decision to submit for publication.

## Data sharing statement

All the data in this study were obtained from publicly available websites like CDC PLACES https://www.cdc.gov/places/index.html, and EJI data were obtained from CDC website https://www.atsdr.cdc.gov/place-health/php/eji/index.html.

## Editor note

The Lancet Group takes a neutral position with respect to territorial claims in published maps and institutional affiliations.

## Declaration of interests

KN has served on the Advisory Boards of Amgen, Regeneron, and Merck Sharp & Dohme; and his research is partly supported by grants from the National Institutes of Health, the Patient-Centered Outcomes Research Institute, Novartis, and Ionis. Other authors declare no competing interests.
