# Supplementary material for: Environmental justice index and prevalence of asthma and COPD in US neighborhoods- a population-based study
Source: Lancet Reg Health Am. 2025 Aug 5;49:101195. doi: 10.1016/j.lana.2025.101195 (PMC12345341; doi:10.1016/j.lana.2025.101195)
Supplement: Supplemental Methods and Tables [file mmc1.docx]

**Supplemental Appendix**

**Table 1. Various indicators of Environmental Justice Index- Social Vulnerability module and Environmental Burden Module.**

| **Overall Environmental Justice Index** | **Social Vulnerability Module** | |
| --- | --- | --- |
|  | Racial/Ethnic minority | - Minority status |
|  | Socio-economic status | - Poverty - No High School Diploma - Unemployment - Housing Tenure - Housing Burdened Lower-Income Households - Lack of Health Insurance - Lack of Broadband access |
|  | Household characteristics | - Age 65 and Older - Age 17 and Younger - Civilians with a Disability - Speak English "Less than Well" |
|  | Housing type | - Group Quarters - Mobile Homes |
|  | **Environmental Burden Module** | |
|  | Air pollution | - Ozone - PM 2.5 - Diesel Particulate matter - Air Toxics cancer risk |
|  | Potentially Hazardous & Toxic sites | - National priority list sites - Toxic Release Inventory sites - Treatment, Storage, and Disposal - Risk Management Plan Sites - Coal Mines - Lead Mines |
|  | Built Environment | - Recreational Parks - Houses Built pre-1980. - Walkability |
|  | Transportation Infrastructure | - High Volume roads - Railways - Airports |
|  | Water pollution | - Impaired Surface water |
|  | **Health Vulnerability Module** | |
|  | Pre-existing Chronic Disease Burden | - Asthma - Cancer - High blood Pressure - Diabetes - Poor Mental Health |

**Outcomes**

A multi-level regression and post-stratification approach was applied to BRFSS and ACS data to compute a detailed probability of having current asthma (reporting 'yes' to both of the questions, "Have you ever been told by a doctor, nurse, or other health professional that you have asthma?" and the question, "Do you still have asthma?"). To compute a detailed probability of adults with COPD who reported having ever been told by a doctor, nurse, or other health professional they had chronic obstructive pulmonary disease (COPD), emphysema, or chronic bronchitis. The probability was then applied to the detailed population estimates at the appropriate geographic level to generate the prevalence. The 95% confidence interval was derived using Monte Carlo simulation.

**Environmental Justice Index Data Sources and Timeframes**

**Background**

The Environmental Justice Index (EJI) represents the first comprehensive nationwide environmental justice assessment tool that examines cumulative environmental and health equity effects at the census tract level. Developed by the Centers for Disease Control and Prevention in collaboration with the Agency for Toxic Substances and Disease Registry, the EJI advances beyond previous screening tools by incorporating multi-dimensional environmental exposures with established social vulnerability measures. This supplemental document provides detailed information on the temporal coverage, data sources, and methodological approaches used in constructing the index components for the 2022 release.

**Data Timeframes and Sources**

**Environmental Burden Module (2014-2022)**

**Air Quality Data (2014-2016)**

Air pollution indicators were derived from multiple Environmental Protection Agency (EPA) monitoring and modeling systems to capture both criteria pollutants and toxic air contaminants. Ground-level ozone and fine particulate matter (PM2.5) concentrations were obtained from EPA's Air Quality System combined with modeled predictions, using 2014-2016 three-year averages to account for meteorological variability and provide stable estimates. These data utilized EPA's Downscaler model and were cross-walked from 2010 to 2020 census tract boundaries. Diesel particulate matter estimates and air toxics cancer risk assessments were derived from EPA's 2014 National Air Toxics Assessment database. The 2014 NATA provides comprehensive estimates of hazardous air pollutants and associated cancer risks across census tracts, representing the most current available modeling framework for toxic air contaminant exposure assessment at the time of EJI 2022 development.

**Hazardous Sites Data (2021)**

Proximity to potentially hazardous facilities and contaminated sites was assessed using multiple EPA databases updated through 2021. National Priorities List sites represent the most severely contaminated locations requiring long-term remedial action under the Superfund program. Toxic Release Inventory facilities include industrial facilities required to report annual releases of specific toxic chemicals, providing insight into ongoing emission sources. Treatment, Storage, and Disposal facilities for hazardous waste were identified through EPA's Resource Conservation and Recovery Act Information system. Risk Management Plan sites encompass facilities using extremely hazardous substances in quantities above regulatory thresholds. Active coal and lead mining operations were identified through the 2021 U.S. Mine Safety and Health Administration's Mine Data Retrieval System, focusing only on active or intermittent operations to reflect current environmental health risks.

**Built Environment Data (2015-2021)**

Built environment indicators captured neighborhood-level factors affecting health through multiple pathways using the most recent available data. Recreational parks access was quantified using the 2020 TomTom MultiNet® Enterprise Dataset, which identifies public green spaces and recreational areas, reflecting the protective effects of natural environments on respiratory health and overall well-being. Housing stock age focused on pre-1980 construction using 2015-2019 American Community Survey data as a proxy for potential lead-based paint exposure and generally poorer indoor environmental quality. Neighborhood walkability metrics incorporated street connectivity, population density, and mixed land use patterns from EPA's 2021 National Walkability Index, recognizing that walkable communities promote physical activity while potentially reducing vehicle emissions exposure.

**Transportation Infrastructure (2020-2021)**

Transportation-related exposures were assessed through proximity to major emission sources using publicly available federal datasets. High-volume roadways were identified through the 2020 TomTom MultiNet® Enterprise Dataset. Active freight and passenger railways were obtained from the 2020 TomTom MultiNet® Enterprise Dataset, excluding abandoned or out-of-service lines. Commercial airports were identified through the 2020 TomTom MultiNet® Enterprise Dataset, focusing on operational airports with at least one runway. These indicators capture exposure to traffic-related air pollutants, noise, and vibration, which have established associations with respiratory health outcomes.

**Water Quality Data (2019)**

Surface water quality was assessed using the 2019 EPA's Watershed Index Online, which integrates multiple water quality parameters including chemical, physical, and biological indicators. Impaired surface water assessments identified water bodies not meeting Clean Water Act standards under Section 303(d), potentially indicating broader environmental degradation affecting community health.

**Social Vulnerability Module (2015-2019)**

The Social Vulnerability Module utilized U.S. Census Bureau American Community Survey 5-year estimates from 2015-2019 to ensure statistical reliability at the census tract level while capturing current demographic and socioeconomic patterns. This timeframe represents stable demographic conditions while providing a baseline for social vulnerability assessment. Racial and ethnic minority status indicators reflect documented health disparities and the effects of structural racism on environmental justice outcomes. Socioeconomic measures include poverty rates (below 200% federal poverty level), educational attainment, unemployment, housing tenure, housing cost burden for lower-income households, health insurance coverage, and internet access. Household characteristics encompass age structure (≥65 years and ≤17 years), disability prevalence, and English language proficiency. Housing type indicators include group quarters residence and mobile home occupancy, reflecting populations with limited capacity to influence environmental decision-making.

**Health Vulnerability Module (2020)**

Health vulnerability data represents 2020 chronic disease prevalence estimates from the CDC's PLACES dataset, providing the most current available chronic disease prevalence estimates at the time of EJI 2022 development.

**Data Integration and Temporal Considerations**

The varying timeframes across EJI components reflect the different data collection and reporting cycles of federal agencies while maintaining analytical validity for cumulative impact assessment. Environmental indicators prioritize the most recent available data (2014-2021) to capture current exposure conditions, while social vulnerability indicators utilize 5-year estimates (2015-2019) to provide stable demographic characterizations with sufficient statistical precision at the census tract level. Health vulnerability data (2020) represents the most current available chronic disease prevalence estimates.

All temporal frameworks overlap sufficiently to support valid cross-module analysis, as both environmental conditions and social vulnerability patterns typically exhibit stability over several-year periods. The geographic scope is limited to the Continental United States (48 states plus District of Columbia) due to data availability constraints for Alaska, Hawaii, Puerto Rico, and other U.S. territories.

**Prevalence of Asthma and COPD stratified by EJI and EBM (****Table S2)**

| **Prevalence per 100,000** | **Prevalence in Mean (SD) as per Environmental Justice Index (EJI)** | | | |
| --- | --- | --- | --- | --- |
|  | **Low** | **Low medium** | **Medium** | **High** |
| **Asthma** | 9684.96(984.99) | 10118.44(1140.65) | 10528.71(1258.91) | 11488.18(1651.79 |
| **COPD** | 5545.72(1818.06) | 6600.76(2394.74) | 7099.251(2459.83) | 8130.06(2733.72) |
|  | **Prevalence in Mean (SD) as per Environmental burden module (EBM)** | | | |
| **Asthma** | 10180.19(1195.04) | 10288.65(1274.56) | 10505.8(1473.711) | 10845.54(1688.11) |
| **COPD** | 6818.99(2469.88) | 6750.36(2412.29) | 6779.39(2498.02) | 7060.31(2793.63) |
|  | **Prevalence in Mean (SD) as per Social Vulnerability module (SVM)** | | | |
| **Asthma** | 9454.97(885.19) | 10088.21(1030.4) | 10671.42(1191.53) | 11675.53(1579.53) |
| **COPD** | 4921.15(1445.57) | 6332.35(1920.2) | 7513.67(2376.30) | 8722.23(2609.43) |

**Interaction Analysis Results and Interpretation**

**Environmental Burden Module Stratified Analysis (Table S4)**

The stratified analysis examining environmental burden effects across different levels of social vulnerability reveals important patterns in how these factors interact to influence respiratory health outcomes. For asthma, the interaction effects are most pronounced in communities with high social vulnerability, where high environmental burden (fourth quartile) shows significant protective associations (RR: 0.977, 95% CI: 0.945-1.009, p<0.001) compared to lower social vulnerability areas. This unexpected finding may reflect complex demographic sorting patterns where the highest social vulnerability communities might have different environmental burden compositions or healthcare access patterns.

For COPD, the interaction patterns are more consistent with the expected synergistic effects. Across all social vulnerability strata, higher environmental burden quartiles show progressively increasing risk ratios, with the strongest effects observed in high social vulnerability communities (fourth quartile EBM: RR: 1.049, 95% CI: 1.016-1.084, p=0.004). The magnitude of environmental burden effects increases with social vulnerability level, supporting the hypothesis that socially vulnerable populations experience greater susceptibility to environmental health impacts.

Prevalence ratios for the low medium/medium/high EBM categories versus the low EBM category within each stratum of SVM

Table S4.

| **Environmental burden module** | | | | |
| --- | --- | --- | --- | --- |
| **Outcomes** | **Low SVM** | **Low medium SVM** | **Medium SVM** | **High SVM** |
| **EBM Fourth Quartile** | | | | |
| Asthma | 1.00 | 0.961 (0.933-0.989)  P=0.007 | 0.979 (0.951-1.009)  P=0.174 | 0.977 (0.945-1.009) P<0.001 |
| COPD | 1.00 | 1.019 (0.989-1.050)  P=0.202 | 1.067 (1.036-1.100)  P<0.001 | 1.049 (1.016-1.084)  P=0.0036 |
| **EBM Third quartile** | | | | |
| Asthma | 1.00 | 1.007 (0.987-1.027)  P=0.474 | 1.027 (1.004-1.049)  P=0.017 | 1.081 (1.053-1.109)  P<0.001 |
| COPD | 1.00 | 1.046 (1.025-1.068)  P<0.001 | 1.095 (1.072-1.119)  P<0.001 | 1.156 (1.128-1.186)  P<0.001 |
| **EBM Second quartile** | | | | |
| Asthma | 1.00 | 1.000 (0.982-1.020)  P=0.926 | 0.995 (0.973-1.017)  P=0.659 | 1.008 (0.982-1.0361)  P=0.514 |
| COPD | 1.00 | 1.065 (1.044-1.086)  P<0.001 | 1.100 (1.076-1.125)  P<0.001 | 1.135 (1.106-1.165)  P<0.001 |
| **EBM First quartile** | | | | |
| Asthma | 1.00 | 1.019 (1.000-1.039) P=0.047 | 0.994 (0.971-1.016)  P=0.612 | 0.995 (0.967-1.025)  P=0.775 |
| COPD | 1.00 | 1.127 (1.104-1.150)  P<0.001 | 1.178 (1.151-1.206)  P<0.001 | 1.212 (1.178-1.247)  P<0.001 |

**Social Vulnerability Module Stratified Analysis (Table S4)**

The complementary analysis examining social vulnerability effects across environmental burden strata demonstrates clearer evidence of synergistic interactions. For both asthma and COPD, the effects of high social vulnerability are amplified in areas with greater environmental burden. In low environmental burden areas, high social vulnerability shows modest effects (asthma RR: 0.994, COPD RR: 1.003), while in high environmental burden areas, the same level of social vulnerability produces substantially larger effect sizes (asthma RR: 1.120, COPD RR: 1.100).

This pattern suggests that environmental exposures may act as effect modifiers, increasing the health impacts of social disadvantage. Communities facing both a high environmental burden and high social vulnerability demonstrate the greatest respiratory disease burden, consistent with environmental justice frameworks emphasizing cumulative impacts.

Prevalence ratios for the low medium/medium/high SVM categories versus the low SVM category within each stratum of EBM Table S4.

| **Social Vulnerability Module** | | | | |
| --- | --- | --- | --- | --- |
| **Outcomes** | **Low EBM** | **Low medium EBM** | **Medium EBM** | **High EBM** |
| **SVM Fourth Quartile** | | | | |
| Asthma | 1.00 | 0.994 (0.968-1.021)  P=0.69 | 1.052 (1.025-1.080)  P<0.001 | 1.120 (1.092-1.149)  P<0.001 |
| COPD | 1.00 | 1.003 (0.978-1.030)  P=0.77 | 1.047 (1.020-1.074)  P<0.001 | 1.100 (1.073-1.128)  P<0.001 |
| **SVM Third quartile** | | | | |
| Asthma | 1.00 | 0.970 (0.952-0.989)  P=0.002 | 1.003 (0.983-1.023)  P=0.736 | 1.083 (1.061-1.105)  P<0.001 |
| COPD | 1.00 | 0.970 (0.953-0.987)  P=0.001 | 0.988 (0.970-1.007)  P=0.222 | 1.064 (1.043-1.085)  P<0.001 |
| **SVM Second quartile** | | | | |
| Asthma | 1.00 | 0.967 (0.948-0.985)  P<0.001 | 0.994 (0.974-1.014)  P=0.574 | 1.039 (1.017-1.061)  P<0.001 |
| COPD | 1.00 | 0.972 (0.955-0.990)  P=0.002 | 0.990 (0.971-1.009)  P=0.344 | 1.036 (1.015-1.057)  P<0.001 |
| **SVM First quartile** | | | | |
| Asthma | 1.00 | 1.012 (0.990-1.034)  P=0.262 | 1.031 (1.008-1.055)  P=0.006 | 1.127 (1.099-1.156)  P<0.001 |
| COPD | 1.00 | 1.028 (1.006-1.050)  P=0.01 | 1.046 (1.023-1.070)  P<0.001 | 1.118 (1.090-1.146)  P<0.001 |

**Statistical Significance and Mechanistic Interpretation**

The interaction terms across multiple models consistently achieve statistical significance (p<0.05), indicating that the combined effects of environmental burden and social vulnerability cannot be explained by their independent contributions alone. However, the observed interactions exhibit variable magnitude and occasionally non-monotonic relationships across exposure strata, suggesting that intervention strategies must account for community-specific risk profiles rather than assuming uniform multiplicative effects.

The biological plausibility of these interactions reflects well-established mechanisms in environmental health research. Environmental exposures, particularly air pollutants, trigger inflammatory cascades and oxidative stress pathways that can exacerbate existing respiratory conditions. Social vulnerability factors may amplify these biological responses through multiple mechanisms: chronic psychosocial stress that dysregulates immune function and increases inflammatory markers; reduced access to preventive healthcare that allows environmental effects to accumulate; poor housing quality that increases both indoor and outdoor pollutant exposure; and limited economic resources that prevent protective behaviors such as air conditioning use during poor air quality days.

The complex interaction patterns observed, including some protective associations in high-burden areas, highlight the importance of comprehensive cumulative risk assessment that accounts for local contextual factors. These findings support the need for place-based interventions that address both exposure reduction and community resilience building, recognizing that environmental and social factors work through interconnected pathways to influence respiratory health outcomes.

**Implications for Environmental Justice Practice**

The significant interactions demonstrate that communities experiencing multiple stressors require integrated intervention approaches. Addressing either environmental or social factors alone may yield suboptimal health improvements compared to comprehensive strategies targeting both domains simultaneously. The non-linear nature of some interactions suggests that community-specific risk assessment and intervention design are essential for effective environmental justice practice.

Study flow chart

**Study Population**

(71,677 US Census Tracts)

**Data Sources**
CDC Environmental Justice Index (2022)
CDC PLACES Dataset (2023)

*Fully adjusted - age and sex composition, healthcare visits, smoking status, and rurality as covariates

**Analysis Groups**
Stratified by EBM Quartiles (Q1-Q4)
- Q1 (lowest burden): n=17,928
- Q2: n=17,926
- Q3: n=17,922
- Q4 (highest burden): n=17,901

n=census tracts

**Statistical Analysis**
Quasi-Poisson Regression Models
- Unadjusted
- Age-category adjusted
- Fully adjusted*

**Analysis Groups**
Stratified by SVM Quartiles (Q1-Q4)
- Q1 (lowest burden): n=17,895
- Q2: n=17,927
- Q3: n=17,928
- Q4 (highest burden): n=17,927

n=census tracts

**Analysis Groups**
Stratified by EJI Quartiles (Q1-Q4)
- Q1 (lowest burden): n=17,920
- Q2: n=17,913
- Q3: n=17,920
- Q4 (highest burden): n=17,924

n=census tracts

**Outcome Measures**
Asthma Prevalence (2020-2021)
COPD Prevalence (2020-2021)

**Primary Exposure Measures**
Environmental Justice Index (EJI)
 Environmental Burden Module (EBM)
 Social Vulnerability Module (SVM)
